# 1-Bromo-1′-(diphenyl­thio­phosphor­yl)­ferrocene

**DOI:** 10.1107/S1600536809036459

**Published:** 2009-09-12

**Authors:** Petr Štěpnička, Jiří Schulz, Ivana Císařová

**Affiliations:** a Department of Inorganic Chemistry, Faculty of Science, Charles University in Prague, Hlavova 2030, 12840 Prague 2, Czech Republic

## Abstract

The title compound, [Fe(C_5_H_4_Br)(C_17_H_14_PS)], crystallizes with two practically undistiguishable mol­ecules in the asymmetric unit, which are related by a non-space-group inversion. The ferrocene-1,1′-diyl units exhibit a regular geometry with negligible tilting and balanced Fe–ring centroid distances, and with the attached substituents assuming conformations close to ideal synclinal eclipsed.

## Related literature

For an overview of the chemistry of ferrocene, see: Štěpnička (2008 ▶); Butler & Davies (1996 ▶). For related structures, see: Fang *et al.* (1995 ▶); Hnetinka *et al.* (2004 ▶); Štěpnička & Císařová (2006*a* ▶,*b* ▶); Labande *et al.* (2007 ▶); Štěpnička *et al.* (2007 ▶).
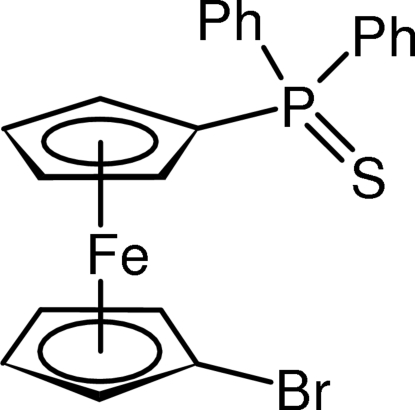


## Experimental

### 

#### Crystal data

[Fe(C_5_H_4_Br)(C_17_H_14_PS)]
*M* *_r_* = 481.15
Monoclinic, 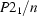

*a* = 13.3590 (7) Å
*b* = 17.3446 (7) Å
*c* = 17.0297 (9) Å
β = 102.460 (2)°
*V* = 3853.0 (3) Å^3^
*Z* = 8
Mo *K*α radiation
μ = 3.05 mm^−1^
*T* = 150 K
0.25 × 0.18 × 0.02 mm

#### Data collection

Nonius KappaCCD diffractometer
Absorption correction: integration (*COLLECT*; Nonius, 2000 ▶) *T*_min_ = 0.540, *T*_max_ = 0.937
53227 measured reflections
6802 independent reflections
4020 reflections with *I* > 2σ(*I*)
*R*_int_ = 0.122

#### Refinement

*R*[*F* ^2^ > 2σ(*F* ^2^)] = 0.077
*wR*(*F* ^2^) = 0.232
*S* = 1.05
6802 reflections
469 parameters
H-atom parameters constrained
Δρ_max_ = 2.19 e Å^−3^
Δρ_min_ = −1.30 e Å^−3^

### 

Data collection: *COLLECT* (Nonius, 2000 ▶); cell refinement: *HKL* *SCALEPACK* (Otwinowski & Minor, 1997 ▶); data reduction: *HKL* *DENZO* (Otwinowski & Minor, 1997 ▶) and *SCALEPACK*; program(s) used to solve structure: *SIR97* (Altomare *et al.*, 1999 ▶); program(s) used to refine structure: *SHELXL97* (Sheldrick, 2008 ▶); molecular graphics: *PLATON* (Spek, 2009 ▶); software used to prepare material for publication: *PLATON*.

## Supplementary Material

Crystal structure: contains datablocks I, global. DOI: 10.1107/S1600536809036459/dn2483sup1.cif

Structure factors: contains datablocks I. DOI: 10.1107/S1600536809036459/dn2483Isup2.hkl

Additional supplementary materials:  crystallographic information; 3D view; checkCIF report

## Figures and Tables

**Fig. 1.**
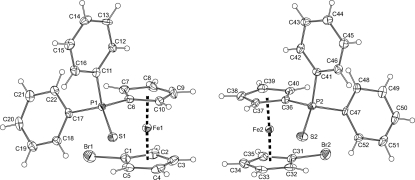
Views of the two independent molecules of the title compound showing the atom numbering scheme and displacement ellipsoids for the non-H atoms at the 30% probability level. Mutual orientation of the molecules does not correspond with that encountered in the crystal.

**Fig. 2.**
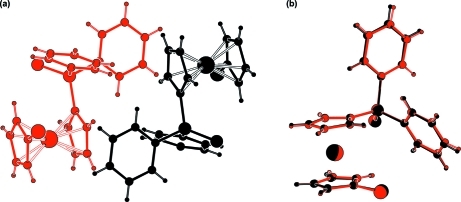
(a) View of the two crystallographically independent molecules illustrating their mutual orientation in the cell (molecule 1 in black, molecule 2 in red). (b) An overlap plot of the independent molecules as generated by PLATON. Inverted molecule 2 (in red) was fitted onto molecule 1 (in black).

## supplementary crystallographic information

### Comment

1'-(Diphenylthiophosphoryl)-1-bromoferrocene, readily accessible by the reaction of 1'-(diphenylphosphino)-1-bromoferrocene with elemental sulfur, can be regarded a protected intermediate suitable for the preparation of functionalized ferrocene phosphines *via* a sequence comprising lithiation, reaction with an appropriate electrophile (Štěpnička, 2008; Butler & Davies, 1996) and, finally, deprotection of the phosphorus group.

The title compound crystallizes with the symmetry of the monoclinic space group *P*2_1_/*n* and two virtually identical molecules in the asymmetric unit (Fig. 1; see also an overlap in Fig. 2) that are related by non-space-group inversion operation. Ferrocene units in the independent molecules have regular geometries, showing negligible tilts (*ca* 2 °) and statistically insignificant variation in the Fe–*Cg* distances (*Cg* denotes a cyclopentadienyl ring centroid; *cf.* Fe–*Cg*: 1.648 (5), 1.643 (5), 1.645 (4), and 1.643 (4) Å for the rings C(1–5), C(6–10), C(31–35), and C(36–40), respectively). Likewise, both molecules assume similar synclinal eclipsed conformation as indicated by the torsion angles Br—*Cg*—*Cg*—P = +75° and -77°, for molecule 1 and 2, respectively. The C—Br and C—P bond lengths compare well with those reported for 1,1'-dibromoferrocene (Hnetinka *et al.*, 2004) or 1'-(diphenylthiophosphoryl)-1-bromoferrocene (Štěpnička & Císařová, 2006*b*), and for 1,1'-bis(diphenylthiophosphoryl)ferrocene (Fang *et al.*, 1995) and 1'-functionalized (diphenylthiophosphoryl)ferrocenes Ph_2_P(S)fcX (fc = ferrocene-1,1'-diyl), where *X* = CHCH_2_ (Štěpnička & Císařová, 2006*a*), CHO, CHCBr_2_ and C≡CH (Štěpnička & Císařová, 2006*b*), CO_2_H (Štěpnička *et al.*, 2007), and CH_2_(C_3_H_3_N_2_Me)^+^ BF_4_^-^ (Labande *et al.*, 2007), respectively.

### Experimental

The title compound was synthesized from the corresponding phosphine and elemental sulfur as follows. 1'-(Diphenylphosphino)-1-bromoferrocene (629 mg, 1.4 mmol; prepared according to Butler & Davies, 1996) and sulfur (64 mg, 2.0 mmol) were mixed with dry toluene (25 ml), and the the resulting solution was heated at reflux under an argon atmosphere for 1 h. The reaction mixture was cooled, filtered and concentrated under reduced pressure to about a half of the initial volume. Slow cooling to -18°C afforded 1'-(diphenylthiophosphoryl)-1-bromoferrocene as an orange microcrystalline solid. Yield: 490 mg (73%).

Characterization data: ^1^H NMR (CDCl_3_): δ 4.16 and 4.38 (2× apparent t, *J* = 1.9 Hz, 2 H, C_5_H_4_Br); 4.49 and 4.53 (2× apparent q, *J* = 2.0 Hz, 2 H, C_5_H_4_P); 7.39–7.75 (m, 10 H, PPh_2_). ^13^C{^1^H} NMR (CDCl_3_): 69.76 and 71.80 (2× s, 2 C, CH of C_5_H_4_Br); 74.92 (d, *J*_PC_ = 12 Hz, 2 C, CH of C_5_H_4_P), 75.37 (d, *J*_PC_ = 10 Hz, 2 C, CH of C_5_H_4_P), 76.70 (d, ^1^*J*_PC_ = 97 Hz, C*_ipso_* of C_5_H_4_P), 77.75 (s, C*_ipso_* of C_5_H_4_Br), 128.27 (d, ^3^*J*_PC_ = 12 Hz, 4 C, CH*_meta_* of PPh_2_), 131.31 (d, ^4^*J*_PC_ = 3 Hz, 2 C, CH*_para_* of PPh_2_), 131.59 (d, ^2^*J*_PC_ = 11 Hz, 4 C, CH*_ortho_* of PPh_2_), 134.28 (d, ^1^*J*_PC_ = 87 Hz, 2 C, C*_ipso_* of PPh_2_). ^31^P{^1^H} NMR (CDCl_3_): δ 41.5 (*s*).

### Refinement

All H atoms were included in their calculated positions and refined as riding atoms with *U*_iso_(H) = 1.2*U*_eq_ of their bonding carbon atom. The unusually high residual electron density can be attributed to an unfavourable crystal shape (thin plates) of the available crystals and their lamellated internal structure, which results in crystal defects (a lower periodicity of the crystal assembly) and, consequently, to the high residual electron density observed.

### Crystal data

**Table:**

[Fe(C_5_H_4_Br)(C_17_H_14_PS)]	*F*(000) = 1936
*M**_r_* = 481.15	*D*_x_ = 1.659 Mg m^−^^3^
Monoclinic, *P*2_1_/*n*	Mo *K*α radiation, λ = 0.71073 Å
Hall symbol: -P 2yn	Cell parameters from 74781 reflections
*a* = 13.3590 (7) Å	θ = 1.0–25.0°
*b* = 17.3446 (7) Å	µ = 3.05 mm^−^^1^
*c* = 17.0297 (9) Å	*T* = 150 K
β = 102.460 (2)°	Plate, orange
*V* = 3853.0 (3) Å^3^	0.25 × 0.18 × 0.02 mm
*Z* = 8	

### Data collection

**Table:**

Nonius KappaCCD diffractometer	6802 independent reflections
Radiation source: fine-focus sealed tube	4020 reflections with *I* > 2σ(*I*)
horizontally mounted graphite crystal	*R*_int_ = 0.122
Detector resolution: 9.091 pixels mm^-1^	θ_max_ = 25.1°, θ_min_ = 1.7°
ω and π scans to fill the Ewald sphere	*h* = −15→15
Absorption correction: integration (*COLLECT*; Nonius, 2000)	*k* = −20→20
*T*_min_ = 0.540, *T*_max_ = 0.937	*l* = −20→20
53227 measured reflections	

### Refinement

**Table:**

Refinement on *F*^2^	Primary atom site location: structure-invariant direct methods
Least-squares matrix: full	Secondary atom site location: difference Fourier map
*R*[*F*^2^ > 2σ(*F*^2^)] = 0.077	Hydrogen site location: inferred from neighbouring sites
*wR*(*F*^2^) = 0.232	H-atom parameters constrained
*S* = 1.05	*w* = 1/[σ^2^(*F*_o_^2^) + (0.1118*P*)^2^ + 21.2954*P*] where *P* = (*F*_o_^2^ + 2*F*_c_^2^)/3
6802 reflections	(Δ/σ)_max_ < 0.001
469 parameters	Δρ_max_ = 2.19 e Å^−^^3^
0 restraints	Δρ_min_ = −1.30 e Å^−^^3^

### Special details

**Table:**

Geometry. All e.s.d.'s (except the e.s.d. in the dihedral angle between two l.s. planes) are estimated using the full covariance matrix. The cell e.s.d.'s are taken into account individually in the estimation of e.s.d.'s in distances, angles and torsion angles; correlations between e.s.d.'s in cell parameters are only used when they are defined by crystal symmetry. An approximate (isotropic) treatment of cell e.s.d.'s is used for estimating e.s.d.'s involving l.s. planes.
Refinement. Refinement of *F*^2^ against all diffractions. The weighted *R*-factor *wR* and goodness of fit *S* are based on *F*^2^, conventional *R*-factors *R* are based on *F*, with *F* set to zero for negative *F*^2^. The threshold expression of *F*^2^ > σ(*F*^2^) is used only for calculating *R*-factors(gt) *etc*. and is not relevant to the choice of reflections for refinement. *R*-factors based on *F*^2^ are statistically about twice as large as those based on *F*, and *R*- factors based on ALL data will be even larger.

### Fractional atomic coordinates and isotropic or equivalent isotropic displacement parameters (Å^2^)

**Table:**

	*x*	*y*	*z*	*U*_iso_*/*U*_eq_	
Br1	0.85670 (9)	0.54776 (7)	0.15983 (7)	0.0553 (4)	
Fe1	0.80463 (9)	0.57655 (7)	0.35241 (8)	0.0282 (3)	
P1	0.72687 (16)	0.39009 (12)	0.36630 (14)	0.0230 (5)	
S1	0.84979 (17)	0.35593 (13)	0.44183 (14)	0.0319 (6)	
C1	0.8910 (7)	0.5779 (6)	0.2679 (6)	0.040 (3)	
C2	0.8859 (7)	0.6525 (5)	0.3000 (6)	0.040 (2)	
H2	0.8597	0.6966	0.2718	0.048*	
C3	0.9281 (7)	0.6474 (6)	0.3827 (7)	0.043 (3)	
H3	0.9350	0.6881	0.4191	0.052*	
C4	0.9582 (7)	0.5711 (6)	0.4016 (7)	0.045 (3)	
H4	0.9885	0.5527	0.4524	0.054*	
C5	0.9349 (7)	0.5267 (6)	0.3305 (7)	0.039 (2)	
H5	0.9462	0.4741	0.3258	0.047*	
C6	0.7056 (6)	0.4930 (5)	0.3685 (6)	0.028 (2)	
C7	0.6582 (6)	0.5407 (5)	0.3007 (6)	0.030 (2)	
H7	0.6329	0.5244	0.2481	0.036*	
C8	0.6581 (8)	0.6174 (5)	0.3304 (7)	0.043 (3)	
H8	0.6352	0.6610	0.3001	0.051*	
C9	0.6993 (8)	0.6153 (5)	0.4144 (7)	0.040 (3)	
H9	0.7048	0.6576	0.4486	0.048*	
C10	0.7310 (7)	0.5391 (5)	0.4388 (6)	0.031 (2)	
H10	0.7620	0.5229	0.4904	0.038*	
C11	0.6117 (6)	0.3477 (5)	0.3868 (5)	0.0246 (19)	
C12	0.5310 (7)	0.3923 (5)	0.4047 (5)	0.028 (2)	
H12	0.5366	0.4457	0.4069	0.033*	
C13	0.4442 (6)	0.3574 (6)	0.4189 (6)	0.033 (2)	
H13	0.3916	0.3874	0.4305	0.039*	
C14	0.4344 (7)	0.2782 (6)	0.4159 (6)	0.039 (2)	
H14	0.3755	0.2551	0.4256	0.046*	
C15	0.5121 (7)	0.2334 (6)	0.3986 (6)	0.040 (2)	
H15	0.5052	0.1800	0.3962	0.048*	
C16	0.6008 (7)	0.2679 (5)	0.3847 (5)	0.033 (2)	
H16	0.6534	0.2373	0.3739	0.039*	
C17	0.7236 (7)	0.3656 (4)	0.2624 (5)	0.026 (2)	
C18	0.8128 (7)	0.3432 (5)	0.2390 (6)	0.031 (2)	
H18	0.8737	0.3391	0.2774	0.038*	
C19	0.8121 (8)	0.3271 (5)	0.1601 (7)	0.042 (3)	
H19	0.8720	0.3122	0.1447	0.051*	
C20	0.7211 (9)	0.3333 (5)	0.1037 (6)	0.041 (3)	
H20	0.7206	0.3235	0.0499	0.049*	
C21	0.6317 (8)	0.3535 (5)	0.1252 (6)	0.037 (2)	
H21	0.5710	0.3566	0.0864	0.044*	
C22	0.6322 (7)	0.3691 (5)	0.2049 (5)	0.029 (2)	
H22	0.5715	0.3821	0.2201	0.035*	
Br2	0.15365 (8)	0.37352 (6)	0.34428 (7)	0.0489 (3)	
Fe2	0.20450 (9)	0.34881 (7)	0.15086 (8)	0.0241 (3)	
P2	0.27899 (16)	0.53407 (12)	0.12992 (14)	0.0236 (5)	
S2	0.15627 (18)	0.56445 (14)	0.05199 (16)	0.0376 (6)	
C31	0.1180 (7)	0.3485 (5)	0.2346 (6)	0.031 (2)	
C32	0.0746 (7)	0.4003 (6)	0.1723 (7)	0.039 (3)	
H32	0.0628	0.4526	0.1778	0.046*	
C33	0.0526 (6)	0.3575 (5)	0.1003 (6)	0.036 (2)	
H33	0.0253	0.3776	0.0495	0.044*	
C34	0.0784 (7)	0.2796 (5)	0.1170 (6)	0.036 (2)	
H34	0.0701	0.2397	0.0796	0.043*	
C35	0.1194 (7)	0.2727 (5)	0.2008 (6)	0.036 (2)	
H35	0.1427	0.2278	0.2286	0.043*	
C36	0.3034 (6)	0.4324 (5)	0.1337 (5)	0.0251 (19)	
C37	0.2800 (7)	0.3822 (5)	0.0653 (6)	0.029 (2)	
H37	0.2496	0.3972	0.0132	0.035*	
C38	0.3102 (7)	0.3067 (5)	0.0895 (6)	0.033 (2)	
H38	0.3038	0.2633	0.0566	0.040*	
C39	0.3531 (7)	0.3090 (5)	0.1747 (6)	0.035 (2)	
H39	0.3792	0.2671	0.2067	0.041*	
C40	0.3487 (6)	0.3862 (5)	0.2016 (6)	0.029 (2)	
H40	0.3712	0.4037	0.2541	0.035*	
C41	0.3951 (6)	0.5776 (5)	0.1109 (5)	0.0230 (19)	
C42	0.4756 (7)	0.5350 (5)	0.0960 (5)	0.031 (2)	
H42	0.4703	0.4815	0.0934	0.037*	
C43	0.5660 (7)	0.5709 (5)	0.0846 (6)	0.033 (2)	
H43	0.6203	0.5418	0.0744	0.039*	
C44	0.5721 (7)	0.6503 (6)	0.0891 (6)	0.039 (2)	
H44	0.6315	0.6750	0.0825	0.047*	
C45	0.4915 (7)	0.6933 (6)	0.1031 (6)	0.036 (2)	
H45	0.4964	0.7468	0.1058	0.043*	
C46	0.4034 (7)	0.6572 (5)	0.1131 (6)	0.033 (2)	
H46	0.3485	0.6867	0.1214	0.039*	
C47	0.2813 (6)	0.5609 (4)	0.2336 (5)	0.0241 (19)	
C48	0.3725 (7)	0.5582 (5)	0.2893 (5)	0.028 (2)	
H48	0.4324	0.5438	0.2737	0.034*	
C49	0.3748 (8)	0.5771 (5)	0.3698 (6)	0.037 (2)	
H49	0.4360	0.5741	0.4081	0.044*	
C50	0.2868 (9)	0.5999 (5)	0.3918 (6)	0.040 (3)	
H50	0.2885	0.6140	0.4448	0.048*	
C51	0.1952 (8)	0.6020 (5)	0.3356 (6)	0.039 (2)	
H51	0.1352	0.6151	0.3517	0.047*	
C52	0.1918 (7)	0.5852 (5)	0.2564 (6)	0.029 (2)	
H52	0.1307	0.5898	0.2183	0.035*	

### Atomic displacement parameters (Å^2^)

**Table:**

	*U*^11^	*U*^22^	*U*^33^	*U*^12^	*U*^13^	*U*^23^
Br1	0.0560 (7)	0.0594 (8)	0.0531 (8)	0.0027 (6)	0.0179 (6)	0.0036 (6)
Fe1	0.0288 (7)	0.0233 (7)	0.0330 (8)	−0.0023 (5)	0.0082 (6)	0.0025 (5)
P1	0.0245 (12)	0.0226 (11)	0.0227 (13)	−0.0005 (9)	0.0071 (10)	−0.0012 (9)
S1	0.0292 (12)	0.0336 (12)	0.0319 (14)	0.0014 (10)	0.0042 (10)	0.0021 (10)
C1	0.042 (6)	0.042 (6)	0.045 (7)	−0.013 (5)	0.029 (5)	−0.009 (5)
C2	0.039 (6)	0.031 (5)	0.051 (7)	−0.007 (4)	0.012 (5)	0.012 (5)
C3	0.031 (5)	0.038 (6)	0.058 (8)	−0.009 (4)	0.003 (5)	−0.002 (5)
C4	0.029 (5)	0.042 (6)	0.058 (8)	−0.005 (4)	0.000 (5)	0.021 (5)
C5	0.028 (5)	0.031 (5)	0.061 (8)	0.003 (4)	0.017 (5)	0.007 (5)
C6	0.021 (4)	0.027 (5)	0.035 (6)	0.002 (4)	0.007 (4)	0.002 (4)
C7	0.023 (5)	0.030 (5)	0.034 (6)	0.006 (4)	−0.002 (4)	0.005 (4)
C8	0.046 (6)	0.018 (5)	0.073 (9)	0.007 (4)	0.032 (6)	0.002 (5)
C9	0.057 (6)	0.022 (5)	0.048 (7)	−0.005 (4)	0.028 (6)	−0.011 (4)
C10	0.033 (5)	0.026 (5)	0.037 (6)	−0.006 (4)	0.015 (4)	−0.004 (4)
C11	0.033 (5)	0.022 (4)	0.020 (5)	−0.005 (4)	0.008 (4)	0.002 (4)
C12	0.034 (5)	0.026 (5)	0.026 (5)	−0.001 (4)	0.013 (4)	−0.001 (4)
C13	0.015 (4)	0.054 (6)	0.032 (6)	0.004 (4)	0.010 (4)	0.003 (5)
C14	0.030 (5)	0.050 (6)	0.036 (6)	−0.018 (5)	0.008 (4)	0.008 (5)
C15	0.045 (6)	0.037 (6)	0.039 (6)	−0.012 (5)	0.013 (5)	0.002 (5)
C16	0.041 (5)	0.037 (5)	0.026 (5)	−0.003 (4)	0.017 (4)	0.001 (4)
C17	0.036 (5)	0.018 (4)	0.025 (5)	−0.002 (4)	0.012 (4)	−0.002 (4)
C18	0.031 (5)	0.030 (5)	0.035 (6)	−0.002 (4)	0.013 (4)	−0.003 (4)
C19	0.047 (6)	0.030 (5)	0.057 (8)	−0.002 (4)	0.027 (6)	−0.008 (5)
C20	0.072 (8)	0.027 (5)	0.027 (6)	−0.003 (5)	0.018 (6)	−0.005 (4)
C21	0.047 (6)	0.029 (5)	0.030 (6)	0.012 (4)	−0.003 (5)	0.001 (4)
C22	0.032 (5)	0.029 (5)	0.023 (5)	0.008 (4)	0.002 (4)	0.002 (4)
Br2	0.0527 (7)	0.0513 (7)	0.0467 (7)	−0.0014 (5)	0.0192 (5)	−0.0049 (5)
Fe2	0.0235 (7)	0.0221 (7)	0.0273 (8)	−0.0006 (5)	0.0070 (5)	0.0010 (5)
P2	0.0236 (11)	0.0238 (11)	0.0244 (13)	0.0009 (9)	0.0074 (10)	0.0022 (9)
S2	0.0301 (13)	0.0393 (14)	0.0406 (16)	0.0027 (10)	0.0013 (11)	0.0100 (11)
C31	0.031 (5)	0.030 (5)	0.038 (6)	−0.005 (4)	0.021 (4)	−0.002 (4)
C32	0.020 (5)	0.033 (5)	0.065 (8)	−0.001 (4)	0.014 (5)	0.007 (5)
C33	0.019 (5)	0.044 (6)	0.045 (7)	−0.004 (4)	0.005 (4)	0.010 (5)
C34	0.033 (5)	0.030 (5)	0.042 (6)	−0.007 (4)	0.006 (4)	0.004 (4)
C35	0.033 (5)	0.029 (5)	0.049 (7)	−0.007 (4)	0.016 (5)	0.002 (4)
C36	0.025 (4)	0.027 (5)	0.026 (5)	−0.003 (4)	0.012 (4)	−0.001 (4)
C37	0.033 (5)	0.035 (5)	0.023 (5)	0.000 (4)	0.014 (4)	−0.004 (4)
C38	0.034 (5)	0.027 (5)	0.043 (7)	0.002 (4)	0.017 (5)	−0.005 (4)
C39	0.028 (5)	0.024 (5)	0.053 (7)	0.003 (4)	0.011 (5)	0.007 (4)
C40	0.021 (5)	0.033 (5)	0.035 (6)	0.003 (4)	0.011 (4)	0.000 (4)
C41	0.023 (4)	0.025 (4)	0.023 (5)	−0.004 (4)	0.007 (4)	0.004 (4)
C42	0.042 (5)	0.027 (5)	0.027 (5)	0.002 (4)	0.016 (4)	−0.001 (4)
C43	0.027 (5)	0.045 (6)	0.027 (6)	0.001 (4)	0.009 (4)	0.003 (4)
C44	0.037 (6)	0.044 (6)	0.042 (6)	−0.009 (5)	0.020 (5)	−0.005 (5)
C45	0.038 (5)	0.035 (5)	0.039 (6)	−0.006 (4)	0.015 (5)	−0.002 (4)
C46	0.034 (5)	0.027 (5)	0.036 (6)	0.003 (4)	0.004 (4)	−0.002 (4)
C47	0.032 (5)	0.020 (4)	0.024 (5)	0.001 (4)	0.015 (4)	−0.008 (4)
C48	0.035 (5)	0.021 (4)	0.030 (6)	0.002 (4)	0.007 (4)	−0.004 (4)
C49	0.053 (6)	0.029 (5)	0.030 (6)	0.003 (4)	0.013 (5)	0.000 (4)
C50	0.073 (8)	0.024 (5)	0.032 (6)	0.002 (5)	0.031 (6)	−0.001 (4)
C51	0.048 (6)	0.029 (5)	0.050 (7)	−0.002 (4)	0.032 (6)	−0.003 (5)
C52	0.026 (5)	0.028 (5)	0.036 (6)	−0.002 (4)	0.014 (4)	−0.001 (4)

### Geometric parameters (Å, °)

**Table:**

Br1—C1	1.872 (10)	Br2—C31	1.877 (10)
Fe1—C6	2.020 (8)	Fe2—C31	2.020 (9)
Fe1—C1	2.030 (9)	Fe2—C36	2.024 (8)
Fe1—C3	2.033 (9)	Fe2—C37	2.027 (9)
Fe1—C2	2.033 (9)	Fe2—C33	2.032 (9)
Fe1—C8	2.038 (10)	Fe2—C40	2.040 (9)
Fe1—C4	2.044 (10)	Fe2—C35	2.043 (9)
Fe1—C10	2.045 (9)	Fe2—C34	2.047 (9)
Fe1—C9	2.046 (10)	Fe2—C32	2.053 (9)
Fe1—C5	2.049 (9)	Fe2—C39	2.057 (9)
Fe1—C7	2.060 (9)	Fe2—C38	2.064 (9)
P1—C11	1.806 (8)	P2—C36	1.792 (8)
P1—C6	1.809 (9)	P2—C41	1.815 (8)
P1—C17	1.811 (9)	P2—C47	1.820 (8)
P1—S1	1.947 (3)	P2—S2	1.946 (3)
C1—C2	1.413 (14)	C31—C32	1.415 (13)
C1—C5	1.415 (14)	C31—C35	1.437 (13)
C2—C3	1.402 (15)	C32—C33	1.409 (14)
C2—H2	0.9300	C32—H32	0.9300
C3—C4	1.401 (14)	C33—C34	1.409 (13)
C3—H3	0.9300	C33—H33	0.9300
C4—C5	1.412 (15)	C34—C35	1.420 (14)
C4—H4	0.9300	C34—H34	0.9300
C5—H5	0.9300	C35—H35	0.9300
C6—C10	1.419 (13)	C36—C40	1.429 (12)
C6—C7	1.451 (12)	C36—C37	1.433 (12)
C7—C8	1.423 (13)	C37—C38	1.406 (12)
C7—H7	0.9300	C37—H37	0.9300
C8—C9	1.418 (15)	C38—C39	1.441 (14)
C8—H8	0.9300	C38—H38	0.9300
C9—C10	1.421 (13)	C39—C40	1.421 (12)
C9—H9	0.9300	C39—H39	0.9300
C10—H10	0.9300	C40—H40	0.9300
C11—C16	1.390 (12)	C41—C42	1.373 (12)
C11—C12	1.412 (12)	C41—C46	1.385 (12)
C12—C13	1.374 (12)	C42—C43	1.409 (12)
C12—H12	0.9300	C42—H42	0.9300
C13—C14	1.379 (13)	C43—C44	1.382 (13)
C13—H13	0.9300	C43—H43	0.9300
C14—C15	1.378 (14)	C44—C45	1.372 (13)
C14—H14	0.9300	C44—H44	0.9300
C15—C16	1.394 (12)	C45—C46	1.376 (13)
C15—H15	0.9300	C45—H45	0.9300
C16—H16	0.9300	C46—H46	0.9300
C17—C18	1.391 (12)	C47—C48	1.374 (12)
C17—C22	1.392 (12)	C47—C52	1.399 (12)
C18—C19	1.371 (14)	C48—C49	1.405 (13)
C18—H18	0.9300	C48—H48	0.9300
C19—C20	1.382 (14)	C49—C50	1.368 (14)
C19—H19	0.9300	C49—H49	0.9300
C20—C21	1.368 (14)	C50—C51	1.381 (15)
C20—H20	0.9300	C50—H50	0.9300
C21—C22	1.383 (13)	C51—C52	1.371 (13)
C21—H21	0.9300	C51—H51	0.9300
C22—H22	0.9300	C52—H52	0.9300
			
C6—Fe1—C1	126.4 (4)	C31—Fe2—C36	126.5 (3)
C6—Fe1—C3	156.7 (4)	C31—Fe2—C37	163.2 (4)
C1—Fe1—C3	67.6 (4)	C36—Fe2—C37	41.4 (3)
C6—Fe1—C2	162.2 (4)	C31—Fe2—C33	68.2 (4)
C1—Fe1—C2	40.7 (4)	C36—Fe2—C33	120.6 (4)
C3—Fe1—C2	40.3 (4)	C37—Fe2—C33	106.3 (4)
C6—Fe1—C8	69.1 (4)	C31—Fe2—C40	109.5 (4)
C1—Fe1—C8	122.4 (4)	C36—Fe2—C40	41.2 (3)
C3—Fe1—C8	122.1 (4)	C37—Fe2—C40	69.1 (4)
C2—Fe1—C8	106.1 (4)	C33—Fe2—C40	157.1 (4)
C6—Fe1—C4	122.7 (4)	C31—Fe2—C35	41.4 (4)
C1—Fe1—C4	67.5 (4)	C36—Fe2—C35	164.1 (4)
C3—Fe1—C4	40.2 (4)	C37—Fe2—C35	153.1 (4)
C2—Fe1—C4	68.0 (4)	C33—Fe2—C35	68.3 (4)
C8—Fe1—C4	158.5 (4)	C40—Fe2—C35	126.0 (4)
C6—Fe1—C10	40.9 (3)	C31—Fe2—C34	68.7 (4)
C1—Fe1—C10	161.7 (4)	C36—Fe2—C34	154.4 (4)
C3—Fe1—C10	120.0 (4)	C37—Fe2—C34	118.1 (4)
C2—Fe1—C10	155.2 (4)	C33—Fe2—C34	40.4 (4)
C8—Fe1—C10	69.5 (4)	C40—Fe2—C34	161.9 (4)
C4—Fe1—C10	106.7 (4)	C35—Fe2—C34	40.6 (4)
C6—Fe1—C9	67.8 (3)	C31—Fe2—C32	40.7 (4)
C1—Fe1—C9	157.1 (4)	C36—Fe2—C32	108.5 (4)
C3—Fe1—C9	106.4 (4)	C37—Fe2—C32	125.0 (4)
C2—Fe1—C9	120.4 (4)	C33—Fe2—C32	40.3 (4)
C8—Fe1—C9	40.6 (4)	C40—Fe2—C32	122.9 (4)
C4—Fe1—C9	123.0 (5)	C35—Fe2—C32	68.9 (4)
C10—Fe1—C9	40.7 (4)	C34—Fe2—C32	68.3 (4)
C6—Fe1—C5	109.2 (4)	C31—Fe2—C39	122.3 (4)
C1—Fe1—C5	40.6 (4)	C36—Fe2—C39	68.7 (3)
C3—Fe1—C5	68.1 (4)	C37—Fe2—C39	68.2 (4)
C2—Fe1—C5	68.8 (4)	C33—Fe2—C39	160.3 (4)
C8—Fe1—C5	158.9 (5)	C40—Fe2—C39	40.6 (3)
C4—Fe1—C5	40.4 (4)	C35—Fe2—C39	107.5 (4)
C10—Fe1—C5	123.8 (4)	C34—Fe2—C39	124.0 (4)
C9—Fe1—C5	159.8 (4)	C32—Fe2—C39	158.0 (4)
C6—Fe1—C7	41.6 (3)	C31—Fe2—C38	156.2 (4)
C1—Fe1—C7	109.4 (4)	C36—Fe2—C38	69.0 (3)
C3—Fe1—C7	159.2 (4)	C37—Fe2—C38	40.2 (3)
C2—Fe1—C7	123.8 (4)	C33—Fe2—C38	122.8 (4)
C8—Fe1—C7	40.6 (4)	C40—Fe2—C38	68.9 (4)
C4—Fe1—C7	159.6 (4)	C35—Fe2—C38	119.0 (4)
C10—Fe1—C7	69.6 (4)	C34—Fe2—C38	105.1 (4)
C9—Fe1—C7	68.0 (4)	C32—Fe2—C38	160.2 (4)
C5—Fe1—C7	124.0 (4)	C39—Fe2—C38	40.9 (4)
C11—P1—C6	104.7 (4)	C36—P2—C41	105.1 (4)
C11—P1—C17	104.6 (4)	C36—P2—C47	104.6 (4)
C6—P1—C17	106.3 (4)	C41—P2—C47	103.3 (4)
C11—P1—S1	112.4 (3)	C36—P2—S2	114.3 (3)
C6—P1—S1	113.6 (3)	C41—P2—S2	113.1 (3)
C17—P1—S1	114.4 (3)	C47—P2—S2	115.3 (3)
C2—C1—C5	109.3 (9)	C32—C31—C35	108.6 (9)
C2—C1—Br1	127.9 (8)	C32—C31—Br2	125.1 (7)
C5—C1—Br1	122.7 (8)	C35—C31—Br2	126.0 (7)
C2—C1—Fe1	69.8 (6)	C32—C31—Fe2	70.9 (5)
C5—C1—Fe1	70.4 (6)	C35—C31—Fe2	70.2 (5)
Br1—C1—Fe1	129.7 (5)	Br2—C31—Fe2	129.9 (5)
C3—C2—C1	106.8 (9)	C33—C32—C31	107.1 (8)
C3—C2—Fe1	69.8 (6)	C33—C32—Fe2	69.0 (5)
C1—C2—Fe1	69.5 (5)	C31—C32—Fe2	68.4 (5)
C3—C2—H2	126.6	C33—C32—H32	126.5
C1—C2—H2	126.6	C31—C32—H32	126.5
Fe1—C2—H2	125.6	Fe2—C32—H32	127.7
C4—C3—C2	108.9 (10)	C32—C33—C34	109.5 (9)
C4—C3—Fe1	70.3 (5)	C32—C33—Fe2	70.7 (5)
C2—C3—Fe1	69.8 (5)	C34—C33—Fe2	70.4 (5)
C4—C3—H3	125.6	C32—C33—H33	125.3
C2—C3—H3	125.6	C34—C33—H33	125.3
Fe1—C3—H3	125.9	Fe2—C33—H33	125.3
C3—C4—C5	108.7 (9)	C33—C34—C35	107.9 (9)
C3—C4—Fe1	69.5 (5)	C33—C34—Fe2	69.2 (5)
C5—C4—Fe1	70.0 (5)	C35—C34—Fe2	69.5 (5)
C3—C4—H4	125.7	C33—C34—H34	126.0
C5—C4—H4	125.7	C35—C34—H34	126.0
Fe1—C4—H4	126.4	Fe2—C34—H34	126.8
C4—C5—C1	106.4 (9)	C34—C35—C31	106.9 (8)
C4—C5—Fe1	69.7 (6)	C34—C35—Fe2	69.8 (5)
C1—C5—Fe1	69.0 (5)	C31—C35—Fe2	68.4 (5)
C4—C5—H5	126.8	C34—C35—H35	126.6
C1—C5—H5	126.8	C31—C35—H35	126.6
Fe1—C5—H5	126.1	Fe2—C35—H35	126.7
C10—C6—C7	109.6 (8)	C40—C36—C37	107.3 (7)
C10—C6—P1	124.3 (7)	C40—C36—P2	128.5 (7)
C7—C6—P1	126.1 (7)	C37—C36—P2	124.2 (7)
C10—C6—Fe1	70.5 (5)	C40—C36—Fe2	70.0 (5)
C7—C6—Fe1	70.7 (5)	C37—C36—Fe2	69.4 (5)
P1—C6—Fe1	126.5 (4)	P2—C36—Fe2	126.2 (4)
C8—C7—C6	106.4 (9)	C38—C37—C36	109.2 (8)
C8—C7—Fe1	68.8 (5)	C38—C37—Fe2	71.3 (5)
C6—C7—Fe1	67.7 (5)	C36—C37—Fe2	69.2 (5)
C8—C7—H7	126.8	C38—C37—H37	125.4
C6—C7—H7	126.8	C36—C37—H37	125.4
Fe1—C7—H7	128.2	Fe2—C37—H37	125.7
C9—C8—C7	107.8 (8)	C37—C38—C39	107.2 (8)
C9—C8—Fe1	70.0 (6)	C37—C38—Fe2	68.5 (5)
C7—C8—Fe1	70.5 (5)	C39—C38—Fe2	69.3 (5)
C9—C8—H8	126.1	C37—C38—H38	126.4
C7—C8—H8	126.1	C39—C38—H38	126.4
Fe1—C8—H8	125.0	Fe2—C38—H38	127.3
C8—C9—C10	110.2 (8)	C40—C39—C38	108.5 (8)
C8—C9—Fe1	69.4 (5)	C40—C39—Fe2	69.0 (5)
C10—C9—Fe1	69.6 (5)	C38—C39—Fe2	69.8 (5)
C8—C9—H9	124.9	C40—C39—H39	125.8
C10—C9—H9	124.9	C38—C39—H39	125.8
Fe1—C9—H9	127.7	Fe2—C39—H39	127.0
C6—C10—C9	106.0 (9)	C39—C40—C36	107.8 (8)
C6—C10—Fe1	68.6 (5)	C39—C40—Fe2	70.4 (5)
C9—C10—Fe1	69.7 (5)	C36—C40—Fe2	68.8 (5)
C6—C10—H10	127.0	C39—C40—H40	126.1
C9—C10—H10	127.0	C36—C40—H40	126.1
Fe1—C10—H10	126.2	Fe2—C40—H40	126.3
C16—C11—C12	118.0 (8)	C42—C41—C46	118.7 (8)
C16—C11—P1	119.3 (7)	C42—C41—P2	122.8 (6)
C12—C11—P1	122.7 (6)	C46—C41—P2	118.5 (7)
C13—C12—C11	120.6 (8)	C41—C42—C43	121.1 (8)
C13—C12—H12	119.7	C41—C42—H42	119.5
C11—C12—H12	119.7	C43—C42—H42	119.5
C12—C13—C14	120.6 (8)	C44—C43—C42	118.4 (8)
C12—C13—H13	119.7	C44—C43—H43	120.8
C14—C13—H13	119.7	C42—C43—H43	120.8
C15—C14—C13	120.0 (8)	C45—C44—C43	120.9 (9)
C15—C14—H14	120.0	C45—C44—H44	119.6
C13—C14—H14	120.0	C43—C44—H44	119.6
C14—C15—C16	120.1 (9)	C44—C45—C46	119.8 (9)
C14—C15—H15	120.0	C44—C45—H45	120.1
C16—C15—H15	120.0	C46—C45—H45	120.1
C11—C16—C15	120.7 (9)	C45—C46—C41	121.1 (9)
C11—C16—H16	119.6	C45—C46—H46	119.4
C15—C16—H16	119.6	C41—C46—H46	119.4
C18—C17—C22	119.1 (8)	C48—C47—C52	120.2 (8)
C18—C17—P1	120.3 (7)	C48—C47—P2	119.2 (6)
C22—C17—P1	120.6 (7)	C52—C47—P2	120.7 (7)
C19—C18—C17	120.9 (9)	C47—C48—C49	119.7 (9)
C19—C18—H18	119.6	C47—C48—H48	120.2
C17—C18—H18	119.6	C49—C48—H48	120.2
C18—C19—C20	119.0 (9)	C50—C49—C48	119.7 (10)
C18—C19—H19	120.5	C50—C49—H49	120.1
C20—C19—H19	120.5	C48—C49—H49	120.1
C21—C20—C19	121.5 (9)	C49—C50—C51	120.2 (9)
C21—C20—H20	119.3	C49—C50—H50	119.9
C19—C20—H20	119.3	C51—C50—H50	119.9
C20—C21—C22	119.6 (9)	C52—C51—C50	120.9 (9)
C20—C21—H21	120.2	C52—C51—H51	119.6
C22—C21—H21	120.2	C50—C51—H51	119.6
C21—C22—C17	120.0 (9)	C51—C52—C47	119.2 (9)
C21—C22—H22	120.0	C51—C52—H52	120.4
C17—C22—H22	120.0	C47—C52—H52	120.4
			
C6—Fe1—C1—C2	163.2 (6)	C36—Fe2—C31—C32	75.2 (7)
C3—Fe1—C1—C2	−38.3 (6)	C37—Fe2—C31—C32	36.2 (15)
C8—Fe1—C1—C2	76.5 (7)	C33—Fe2—C31—C32	−37.4 (6)
C4—Fe1—C1—C2	−81.9 (6)	C40—Fe2—C31—C32	118.2 (6)
C10—Fe1—C1—C2	−156.7 (11)	C35—Fe2—C31—C32	−118.9 (8)
C9—Fe1—C1—C2	40.5 (13)	C34—Fe2—C31—C32	−81.0 (6)
C5—Fe1—C1—C2	−120.3 (9)	C39—Fe2—C31—C32	161.4 (5)
C7—Fe1—C1—C2	119.6 (6)	C38—Fe2—C31—C32	−160.0 (9)
C6—Fe1—C1—C5	−76.5 (7)	C36—Fe2—C31—C35	−165.9 (5)
C3—Fe1—C1—C5	82.0 (6)	C37—Fe2—C31—C35	155.1 (12)
C2—Fe1—C1—C5	120.3 (9)	C33—Fe2—C31—C35	81.5 (6)
C8—Fe1—C1—C5	−163.2 (6)	C40—Fe2—C31—C35	−122.9 (6)
C4—Fe1—C1—C5	38.3 (6)	C34—Fe2—C31—C35	37.9 (5)
C10—Fe1—C1—C5	−36.4 (15)	C32—Fe2—C31—C35	118.9 (8)
C9—Fe1—C1—C5	160.8 (10)	C39—Fe2—C31—C35	−79.7 (6)
C7—Fe1—C1—C5	−120.1 (6)	C38—Fe2—C31—C35	−41.1 (12)
C6—Fe1—C1—Br1	40.1 (9)	C36—Fe2—C31—Br2	−45.0 (8)
C3—Fe1—C1—Br1	−161.3 (8)	C37—Fe2—C31—Br2	−84.1 (15)
C2—Fe1—C1—Br1	−123.0 (10)	C33—Fe2—C31—Br2	−157.7 (7)
C8—Fe1—C1—Br1	−46.6 (8)	C40—Fe2—C31—Br2	−2.0 (7)
C4—Fe1—C1—Br1	155.0 (8)	C35—Fe2—C31—Br2	120.8 (9)
C10—Fe1—C1—Br1	80.3 (15)	C34—Fe2—C31—Br2	158.7 (7)
C9—Fe1—C1—Br1	−82.5 (12)	C32—Fe2—C31—Br2	−120.2 (9)
C5—Fe1—C1—Br1	116.7 (9)	C39—Fe2—C31—Br2	41.2 (7)
C7—Fe1—C1—Br1	−3.4 (8)	C38—Fe2—C31—Br2	79.8 (11)
C5—C1—C2—C3	0.7 (11)	C35—C31—C32—C33	−2.0 (10)
Br1—C1—C2—C3	−174.7 (7)	Br2—C31—C32—C33	−175.8 (6)
Fe1—C1—C2—C3	60.2 (7)	Fe2—C31—C32—C33	58.4 (6)
C5—C1—C2—Fe1	−59.5 (7)	C35—C31—C32—Fe2	−60.3 (6)
Br1—C1—C2—Fe1	125.1 (8)	Br2—C31—C32—Fe2	125.9 (7)
C6—Fe1—C2—C3	−167.5 (11)	C31—Fe2—C32—C33	−119.3 (8)
C1—Fe1—C2—C3	−117.7 (9)	C36—Fe2—C32—C33	115.8 (6)
C8—Fe1—C2—C3	121.0 (6)	C37—Fe2—C32—C33	72.7 (7)
C4—Fe1—C2—C3	−37.1 (6)	C40—Fe2—C32—C33	159.0 (5)
C10—Fe1—C2—C3	45.1 (12)	C35—Fe2—C32—C33	−81.0 (6)
C9—Fe1—C2—C3	79.3 (7)	C34—Fe2—C32—C33	−37.2 (6)
C5—Fe1—C2—C3	−80.7 (7)	C39—Fe2—C32—C33	−165.4 (9)
C7—Fe1—C2—C3	161.7 (6)	C38—Fe2—C32—C33	36.6 (13)
C6—Fe1—C2—C1	−49.8 (14)	C36—Fe2—C32—C31	−124.9 (5)
C3—Fe1—C2—C1	117.7 (9)	C37—Fe2—C32—C31	−168.0 (5)
C8—Fe1—C2—C1	−121.3 (7)	C33—Fe2—C32—C31	119.3 (8)
C4—Fe1—C2—C1	80.6 (7)	C40—Fe2—C32—C31	−81.7 (6)
C10—Fe1—C2—C1	162.8 (9)	C35—Fe2—C32—C31	38.4 (5)
C9—Fe1—C2—C1	−163.0 (6)	C34—Fe2—C32—C31	82.1 (6)
C5—Fe1—C2—C1	37.1 (6)	C39—Fe2—C32—C31	−46.1 (12)
C7—Fe1—C2—C1	−80.5 (7)	C38—Fe2—C32—C31	155.9 (9)
C1—C2—C3—C4	−0.3 (11)	C31—C32—C33—C34	1.9 (10)
Fe1—C2—C3—C4	59.7 (7)	Fe2—C32—C33—C34	59.9 (6)
C1—C2—C3—Fe1	−60.0 (6)	C31—C32—C33—Fe2	−58.0 (6)
C6—Fe1—C3—C4	50.6 (13)	C31—Fe2—C33—C32	37.7 (6)
C1—Fe1—C3—C4	−81.2 (7)	C36—Fe2—C33—C32	−82.7 (6)
C2—Fe1—C3—C4	−119.8 (9)	C37—Fe2—C33—C32	−125.5 (6)
C8—Fe1—C3—C4	163.7 (7)	C40—Fe2—C33—C32	−50.8 (12)
C10—Fe1—C3—C4	80.2 (8)	C35—Fe2—C33—C32	82.5 (6)
C9—Fe1—C3—C4	122.2 (7)	C34—Fe2—C33—C32	120.0 (8)
C5—Fe1—C3—C4	−37.2 (7)	C39—Fe2—C33—C32	163.8 (10)
C7—Fe1—C3—C4	−166.9 (11)	C38—Fe2—C33—C32	−166.1 (5)
C6—Fe1—C3—C2	170.4 (8)	C31—Fe2—C33—C34	−82.3 (6)
C1—Fe1—C3—C2	38.6 (6)	C36—Fe2—C33—C34	157.2 (6)
C8—Fe1—C3—C2	−76.5 (7)	C37—Fe2—C33—C34	114.5 (6)
C4—Fe1—C3—C2	119.8 (9)	C40—Fe2—C33—C34	−170.8 (9)
C10—Fe1—C3—C2	−159.9 (6)	C35—Fe2—C33—C34	−37.5 (6)
C9—Fe1—C3—C2	−118.0 (6)	C32—Fe2—C33—C34	−120.0 (8)
C5—Fe1—C3—C2	82.6 (7)	C39—Fe2—C33—C34	43.8 (14)
C7—Fe1—C3—C2	−47.1 (15)	C38—Fe2—C33—C34	73.9 (7)
C2—C3—C4—C5	−0.2 (11)	C32—C33—C34—C35	−1.1 (10)
Fe1—C3—C4—C5	59.2 (7)	Fe2—C33—C34—C35	58.9 (6)
C2—C3—C4—Fe1	−59.4 (7)	C32—C33—C34—Fe2	−60.1 (6)
C6—Fe1—C4—C3	−158.8 (6)	C31—Fe2—C34—C33	81.0 (6)
C1—Fe1—C4—C3	81.4 (7)	C36—Fe2—C34—C33	−50.5 (11)
C2—Fe1—C4—C3	37.3 (7)	C37—Fe2—C34—C33	−82.1 (7)
C8—Fe1—C4—C3	−40.5 (15)	C40—Fe2—C34—C33	168.5 (11)
C10—Fe1—C4—C3	−117.0 (7)	C35—Fe2—C34—C33	119.6 (8)
C9—Fe1—C4—C3	−75.6 (8)	C32—Fe2—C34—C33	37.1 (6)
C5—Fe1—C4—C3	120.0 (9)	C39—Fe2—C34—C33	−163.6 (6)
C7—Fe1—C4—C3	166.6 (11)	C38—Fe2—C34—C33	−123.2 (6)
C6—Fe1—C4—C5	81.3 (7)	C31—Fe2—C34—C35	−38.6 (6)
C1—Fe1—C4—C5	−38.6 (6)	C36—Fe2—C34—C35	−170.0 (7)
C3—Fe1—C4—C5	−120.0 (9)	C37—Fe2—C34—C35	158.4 (5)
C2—Fe1—C4—C5	−82.7 (6)	C33—Fe2—C34—C35	−119.6 (8)
C8—Fe1—C4—C5	−160.5 (10)	C40—Fe2—C34—C35	49.0 (15)
C10—Fe1—C4—C5	123.0 (6)	C32—Fe2—C34—C35	−82.5 (6)
C9—Fe1—C4—C5	164.5 (6)	C39—Fe2—C34—C35	76.8 (7)
C7—Fe1—C4—C5	46.7 (15)	C38—Fe2—C34—C35	117.2 (6)
C3—C4—C5—C1	0.6 (11)	C33—C34—C35—C31	−0.1 (10)
Fe1—C4—C5—C1	59.5 (6)	Fe2—C34—C35—C31	58.6 (6)
C3—C4—C5—Fe1	−58.9 (7)	C33—C34—C35—Fe2	−58.7 (6)
C2—C1—C5—C4	−0.8 (11)	C32—C31—C35—C34	1.3 (10)
Br1—C1—C5—C4	174.9 (7)	Br2—C31—C35—C34	175.0 (6)
Fe1—C1—C5—C4	−59.9 (7)	Fe2—C31—C35—C34	−59.5 (6)
C2—C1—C5—Fe1	59.1 (7)	C32—C31—C35—Fe2	60.8 (6)
Br1—C1—C5—Fe1	−125.2 (7)	Br2—C31—C35—Fe2	−125.5 (7)
C6—Fe1—C5—C4	−118.3 (6)	C31—Fe2—C35—C34	118.5 (8)
C1—Fe1—C5—C4	117.7 (8)	C36—Fe2—C35—C34	164.2 (12)
C3—Fe1—C5—C4	37.0 (6)	C37—Fe2—C35—C34	−45.9 (11)
C2—Fe1—C5—C4	80.6 (6)	C33—Fe2—C35—C34	37.4 (6)
C8—Fe1—C5—C4	160.2 (9)	C40—Fe2—C35—C34	−163.1 (5)
C10—Fe1—C5—C4	−75.2 (7)	C32—Fe2—C35—C34	80.8 (6)
C9—Fe1—C5—C4	−40.6 (13)	C39—Fe2—C35—C34	−122.2 (6)
C7—Fe1—C5—C4	−162.2 (5)	C38—Fe2—C35—C34	−79.1 (6)
C6—Fe1—C5—C1	124.0 (6)	C36—Fe2—C35—C31	45.7 (15)
C3—Fe1—C5—C1	−80.7 (6)	C37—Fe2—C35—C31	−164.4 (7)
C2—Fe1—C5—C1	−37.2 (6)	C33—Fe2—C35—C31	−81.2 (6)
C8—Fe1—C5—C1	42.5 (13)	C40—Fe2—C35—C31	78.4 (6)
C4—Fe1—C5—C1	−117.7 (8)	C34—Fe2—C35—C31	−118.5 (8)
C10—Fe1—C5—C1	167.1 (5)	C32—Fe2—C35—C31	−37.7 (6)
C9—Fe1—C5—C1	−158.3 (10)	C39—Fe2—C35—C31	119.3 (6)
C7—Fe1—C5—C1	80.1 (6)	C38—Fe2—C35—C31	162.4 (5)
C11—P1—C6—C10	88.7 (8)	C41—P2—C36—C40	89.0 (8)
C17—P1—C6—C10	−161.0 (7)	C47—P2—C36—C40	−19.5 (9)
S1—P1—C6—C10	−34.3 (8)	S2—P2—C36—C40	−146.5 (7)
C11—P1—C6—C7	−89.5 (8)	C41—P2—C36—C37	−90.4 (8)
C17—P1—C6—C7	20.8 (8)	C47—P2—C36—C37	161.1 (7)
S1—P1—C6—C7	147.5 (7)	S2—P2—C36—C37	34.1 (8)
C11—P1—C6—Fe1	178.8 (5)	C41—P2—C36—Fe2	−178.7 (5)
C17—P1—C6—Fe1	−70.8 (6)	C47—P2—C36—Fe2	72.8 (6)
S1—P1—C6—Fe1	55.9 (6)	S2—P2—C36—Fe2	−54.2 (6)
C1—Fe1—C6—C10	162.0 (6)	C31—Fe2—C36—C40	77.6 (6)
C3—Fe1—C6—C10	41.0 (12)	C37—Fe2—C36—C40	−118.4 (7)
C2—Fe1—C6—C10	−159.8 (11)	C33—Fe2—C36—C40	161.8 (5)
C8—Fe1—C6—C10	−82.5 (6)	C35—Fe2—C36—C40	41.5 (15)
C4—Fe1—C6—C10	77.3 (7)	C34—Fe2—C36—C40	−162.7 (8)
C9—Fe1—C6—C10	−38.7 (6)	C32—Fe2—C36—C40	119.2 (6)
C5—Fe1—C6—C10	119.9 (6)	C39—Fe2—C36—C40	−37.6 (5)
C7—Fe1—C6—C10	−120.0 (7)	C38—Fe2—C36—C40	−81.6 (5)
C1—Fe1—C6—C7	−78.0 (7)	C31—Fe2—C36—C37	−164.0 (5)
C3—Fe1—C6—C7	161.0 (9)	C33—Fe2—C36—C37	−79.8 (6)
C2—Fe1—C6—C7	−39.7 (14)	C40—Fe2—C36—C37	118.4 (7)
C8—Fe1—C6—C7	37.5 (6)	C35—Fe2—C36—C37	159.9 (12)
C4—Fe1—C6—C7	−162.7 (6)	C34—Fe2—C36—C37	−44.3 (10)
C10—Fe1—C6—C7	120.0 (7)	C32—Fe2—C36—C37	−122.4 (6)
C9—Fe1—C6—C7	81.3 (6)	C39—Fe2—C36—C37	80.8 (6)
C5—Fe1—C6—C7	−120.0 (6)	C38—Fe2—C36—C37	36.8 (5)
C1—Fe1—C6—P1	43.2 (8)	C31—Fe2—C36—P2	−46.0 (8)
C3—Fe1—C6—P1	−77.9 (12)	C37—Fe2—C36—P2	118.0 (8)
C2—Fe1—C6—P1	81.4 (13)	C33—Fe2—C36—P2	38.2 (8)
C8—Fe1—C6—P1	158.6 (7)	C40—Fe2—C36—P2	−123.6 (8)
C4—Fe1—C6—P1	−41.6 (8)	C35—Fe2—C36—P2	−82.1 (14)
C10—Fe1—C6—P1	−118.8 (8)	C34—Fe2—C36—P2	73.7 (11)
C9—Fe1—C6—P1	−157.5 (7)	C32—Fe2—C36—P2	−4.4 (7)
C5—Fe1—C6—P1	1.1 (7)	C39—Fe2—C36—P2	−161.2 (7)
C7—Fe1—C6—P1	121.1 (9)	C38—Fe2—C36—P2	154.7 (7)
C10—C6—C7—C8	1.8 (10)	C40—C36—C37—C38	−0.3 (9)
P1—C6—C7—C8	−179.8 (7)	P2—C36—C37—C38	179.2 (6)
Fe1—C6—C7—C8	−58.2 (6)	Fe2—C36—C37—C38	−60.3 (6)
C10—C6—C7—Fe1	60.0 (6)	C40—C36—C37—Fe2	60.0 (6)
P1—C6—C7—Fe1	−121.6 (7)	P2—C36—C37—Fe2	−120.5 (6)
C6—Fe1—C7—C8	119.1 (8)	C31—Fe2—C37—C38	170.0 (12)
C1—Fe1—C7—C8	−117.5 (6)	C36—Fe2—C37—C38	120.0 (8)
C3—Fe1—C7—C8	−39.7 (14)	C33—Fe2—C37—C38	−121.9 (6)
C2—Fe1—C7—C8	−74.5 (7)	C40—Fe2—C37—C38	81.7 (6)
C4—Fe1—C7—C8	165.1 (12)	C35—Fe2—C37—C38	−48.0 (10)
C10—Fe1—C7—C8	81.9 (6)	C34—Fe2—C37—C38	−79.9 (6)
C9—Fe1—C7—C8	38.2 (6)	C32—Fe2—C37—C38	−162.0 (6)
C5—Fe1—C7—C8	−160.3 (6)	C39—Fe2—C37—C38	38.0 (5)
C1—Fe1—C7—C6	123.4 (6)	C31—Fe2—C37—C36	49.9 (15)
C3—Fe1—C7—C6	−158.8 (11)	C33—Fe2—C37—C36	118.0 (5)
C2—Fe1—C7—C6	166.4 (5)	C40—Fe2—C37—C36	−38.3 (5)
C8—Fe1—C7—C6	−119.1 (8)	C35—Fe2—C37—C36	−168.0 (8)
C4—Fe1—C7—C6	46.0 (14)	C34—Fe2—C37—C36	160.0 (5)
C10—Fe1—C7—C6	−37.2 (5)	C32—Fe2—C37—C36	78.0 (6)
C9—Fe1—C7—C6	−80.9 (6)	C39—Fe2—C37—C36	−82.0 (5)
C5—Fe1—C7—C6	80.6 (6)	C38—Fe2—C37—C36	−120.0 (8)
C6—C7—C8—C9	−2.9 (10)	C36—C37—C38—C39	0.3 (10)
Fe1—C7—C8—C9	−60.3 (7)	Fe2—C37—C38—C39	−58.7 (6)
C6—C7—C8—Fe1	57.4 (6)	C36—C37—C38—Fe2	59.0 (6)
C6—Fe1—C8—C9	79.8 (6)	C31—Fe2—C38—C37	−172.8 (8)
C1—Fe1—C8—C9	−159.5 (5)	C36—Fe2—C38—C37	−37.9 (5)
C3—Fe1—C8—C9	−77.3 (7)	C33—Fe2—C38—C37	75.7 (6)
C2—Fe1—C8—C9	−118.2 (6)	C40—Fe2—C38—C37	−82.1 (6)
C4—Fe1—C8—C9	−47.6 (13)	C35—Fe2—C38—C37	157.4 (5)
C10—Fe1—C8—C9	36.0 (5)	C34—Fe2—C38—C37	115.9 (6)
C5—Fe1—C8—C9	169.2 (9)	C32—Fe2—C38—C37	48.4 (13)
C7—Fe1—C8—C9	118.3 (8)	C39—Fe2—C38—C37	−119.2 (7)
C6—Fe1—C8—C7	−38.4 (6)	C31—Fe2—C38—C39	−53.6 (11)
C1—Fe1—C8—C7	82.2 (7)	C36—Fe2—C38—C39	81.3 (5)
C3—Fe1—C8—C7	164.5 (6)	C37—Fe2—C38—C39	119.2 (7)
C2—Fe1—C8—C7	123.5 (6)	C33—Fe2—C38—C39	−165.0 (5)
C4—Fe1—C8—C7	−165.9 (10)	C40—Fe2—C38—C39	37.1 (5)
C10—Fe1—C8—C7	−82.2 (6)	C35—Fe2—C38—C39	−83.4 (6)
C9—Fe1—C8—C7	−118.3 (8)	C34—Fe2—C38—C39	−124.9 (5)
C5—Fe1—C8—C7	50.9 (13)	C32—Fe2—C38—C39	167.6 (10)
C7—C8—C9—C10	3.0 (11)	C37—C38—C39—C40	−0.1 (10)
Fe1—C8—C9—C10	−57.7 (7)	Fe2—C38—C39—C40	−58.3 (6)
C7—C8—C9—Fe1	60.7 (6)	C37—C38—C39—Fe2	58.2 (6)
C6—Fe1—C9—C8	−83.3 (6)	C31—Fe2—C39—C40	−82.4 (6)
C1—Fe1—C9—C8	49.6 (12)	C36—Fe2—C39—C40	38.1 (5)
C3—Fe1—C9—C8	120.5 (6)	C37—Fe2—C39—C40	82.8 (6)
C2—Fe1—C9—C8	79.0 (6)	C33—Fe2—C39—C40	160.2 (10)
C4—Fe1—C9—C8	161.2 (5)	C35—Fe2—C39—C40	−125.5 (6)
C10—Fe1—C9—C8	−122.2 (8)	C34—Fe2—C39—C40	−167.1 (5)
C5—Fe1—C9—C8	−168.7 (10)	C32—Fe2—C39—C40	−48.7 (12)
C7—Fe1—C9—C8	−38.2 (5)	C38—Fe2—C39—C40	120.2 (8)
C6—Fe1—C9—C10	38.9 (5)	C31—Fe2—C39—C38	157.4 (5)
C1—Fe1—C9—C10	171.8 (9)	C36—Fe2—C39—C38	−82.0 (5)
C3—Fe1—C9—C10	−117.3 (6)	C37—Fe2—C39—C38	−37.3 (5)
C2—Fe1—C9—C10	−158.8 (6)	C33—Fe2—C39—C38	40.0 (13)
C8—Fe1—C9—C10	122.2 (8)	C40—Fe2—C39—C38	−120.2 (8)
C4—Fe1—C9—C10	−76.6 (6)	C35—Fe2—C39—C38	114.4 (6)
C5—Fe1—C9—C10	−46.5 (13)	C34—Fe2—C39—C38	72.7 (6)
C7—Fe1—C9—C10	84.0 (6)	C32—Fe2—C39—C38	−168.9 (9)
C7—C6—C10—C9	0.0 (10)	C38—C39—C40—C36	−0.1 (10)
P1—C6—C10—C9	−178.5 (6)	Fe2—C39—C40—C36	−58.8 (6)
Fe1—C6—C10—C9	60.1 (6)	C38—C39—C40—Fe2	58.8 (6)
C7—C6—C10—Fe1	−60.1 (6)	C37—C36—C40—C39	0.2 (9)
P1—C6—C10—Fe1	121.5 (7)	P2—C36—C40—C39	−179.3 (6)
C8—C9—C10—C6	−1.8 (10)	Fe2—C36—C40—C39	59.8 (6)
Fe1—C9—C10—C6	−59.4 (6)	C37—C36—C40—Fe2	−59.6 (6)
C8—C9—C10—Fe1	57.5 (7)	P2—C36—C40—Fe2	120.9 (7)
C1—Fe1—C10—C6	−52.5 (14)	C31—Fe2—C40—C39	117.3 (6)
C3—Fe1—C10—C6	−162.6 (5)	C36—Fe2—C40—C39	−119.1 (8)
C2—Fe1—C10—C6	165.4 (8)	C37—Fe2—C40—C39	−80.6 (6)
C8—Fe1—C10—C6	81.3 (6)	C33—Fe2—C40—C39	−162.8 (10)
C4—Fe1—C10—C6	−121.0 (6)	C35—Fe2—C40—C39	73.9 (7)
C9—Fe1—C10—C6	117.4 (8)	C34—Fe2—C40—C39	36.5 (15)
C5—Fe1—C10—C6	−80.2 (6)	C32—Fe2—C40—C39	160.4 (6)
C7—Fe1—C10—C6	37.8 (5)	C38—Fe2—C40—C39	−37.4 (6)
C6—Fe1—C10—C9	−117.4 (8)	C31—Fe2—C40—C36	−123.6 (5)
C1—Fe1—C10—C9	−169.9 (12)	C37—Fe2—C40—C36	38.6 (5)
C3—Fe1—C10—C9	80.0 (7)	C33—Fe2—C40—C36	−43.7 (12)
C2—Fe1—C10—C9	48.1 (12)	C35—Fe2—C40—C36	−167.0 (5)
C8—Fe1—C10—C9	−36.0 (6)	C34—Fe2—C40—C36	155.6 (11)
C4—Fe1—C10—C9	121.6 (6)	C32—Fe2—C40—C36	−80.5 (6)
C5—Fe1—C10—C9	162.4 (6)	C39—Fe2—C40—C36	119.1 (8)
C7—Fe1—C10—C9	−79.5 (6)	C38—Fe2—C40—C36	81.7 (5)
C6—P1—C11—C16	176.3 (7)	C36—P2—C41—C42	5.4 (9)
C17—P1—C11—C16	64.6 (8)	C47—P2—C41—C42	114.8 (8)
S1—P1—C11—C16	−60.0 (8)	S2—P2—C41—C42	−119.9 (7)
C6—P1—C11—C12	−3.4 (8)	C36—P2—C41—C46	−173.4 (7)
C17—P1—C11—C12	−115.1 (8)	C47—P2—C41—C46	−64.0 (8)
S1—P1—C11—C12	120.3 (7)	S2—P2—C41—C46	61.3 (8)
C16—C11—C12—C13	−0.5 (13)	C46—C41—C42—C43	1.3 (13)
P1—C11—C12—C13	179.1 (7)	P2—C41—C42—C43	−177.5 (7)
C11—C12—C13—C14	0.1 (14)	C41—C42—C43—C44	0.2 (14)
C12—C13—C14—C15	0.0 (14)	C42—C43—C44—C45	−0.9 (15)
C13—C14—C15—C16	0.5 (15)	C43—C44—C45—C46	0.1 (15)
C12—C11—C16—C15	1.0 (13)	C44—C45—C46—C41	1.4 (15)
P1—C11—C16—C15	−178.7 (7)	C42—C41—C46—C45	−2.1 (14)
C14—C15—C16—C11	−1.0 (14)	P2—C41—C46—C45	176.8 (7)
C11—P1—C17—C18	−139.3 (7)	C36—P2—C47—C48	68.5 (7)
C6—P1—C17—C18	110.3 (7)	C41—P2—C47—C48	−41.2 (8)
S1—P1—C17—C18	−15.9 (8)	S2—P2—C47—C48	−165.1 (6)
C11—P1—C17—C22	41.0 (8)	C36—P2—C47—C52	−112.4 (7)
C6—P1—C17—C22	−69.4 (7)	C41—P2—C47—C52	137.9 (7)
S1—P1—C17—C22	164.4 (6)	S2—P2—C47—C52	14.0 (8)
C22—C17—C18—C19	2.0 (13)	C52—C47—C48—C49	2.3 (12)
P1—C17—C18—C19	−177.7 (7)	P2—C47—C48—C49	−178.6 (6)
C17—C18—C19—C20	−0.1 (14)	C47—C48—C49—C50	−1.6 (13)
C18—C19—C20—C21	−1.4 (14)	C48—C49—C50—C51	2.0 (13)
C19—C20—C21—C22	0.9 (14)	C49—C50—C51—C52	−3.2 (14)
C20—C21—C22—C17	1.1 (13)	C50—C51—C52—C47	3.9 (13)
C18—C17—C22—C21	−2.5 (12)	C48—C47—C52—C51	−3.5 (12)
P1—C17—C22—C21	177.2 (7)	P2—C47—C52—C51	177.4 (7)
